# Who Speaks for the Dead? Of Communities and Stewardship in Legacy Collections of Human Remains

**DOI:** 10.1002/ajpa.70216

**Published:** 2026-03-10

**Authors:** Fatimah L. C. Jackson, Benjamin M. Auerbach

**Affiliations:** ^1^ Department of Biology Howard University Washington DC USA; ^2^ Department of Ecology and Evolutionary Biology The University of Tennessee Knoxville Tennessee USA; ^3^ Department of Anthropology The University of Tennessee Knoxville Tennessee USA

**Keywords:** AABA task force, bioethics, human tissues, skeletal biology

In this issue of the *American Journal of Biological Anthropology*, we present the Recommendations of the AABA Presidential Task Force on the Ethical Study of Human Remains (Auerbach et al. 2026), which we co‐chaired starting in the summer of 2021. It has been an honor for us to have been entrusted to lead this endeavor. The Recommendations reflect the deliberations of the members of the Task Force based on the feedback we received from over 3254 self‐identified members of the African American community, hundreds of biological anthropologists (including members of the AABA Executive Committee), and many conversations with members of various underrepresented descendant communities, collections managers, curators, the leadership of sibling associations, journal editors, grant agency officials, and members of similar task forces in other anthropological associations and the American Association for Anatomy.

A guiding principle for the members of the Task Force from the outset was that we needed data on which to make recommendations. While we on the Task Force had decades of experience working with human tissues, both in legacy collections and from living participants, we did not want to set out recommendations from our limited experiences and biases. The Task Force determined that the most optimal and novel approach would be to systematically query representative members of descendant communities—the individuals most impacted and least consulted in legacy collections—and biological anthropologists to determine the perspectives of both, to identify areas of congruence, and then to propose possible solutions to the dilemmas posed in the study of human remains stored in scientific laboratories and museums. With thousands of descendant communities, complex histories of how collections were created, as well as different ethical and legal standards across the globe, we knew that we would have to focus on one or two descendant communities for the purposes of creating a workable framework. We understood that this framework would have to provide guidance and executable actions informed by the information we collected while remaining adaptable and flexible.

As we explain in the Recommendations, we chose to engage in conversations with African Americans because of the volume of individuals whose remains are in legacy collections and the urgency of addressing the ethics of their future in these collections. We were motivated by the revelation of the use of recent historical skeletal remains from the 1985 MOVE Bombing in Philadelphia in research and teaching without the permission of either the biological relatives of these individuals or of the larger descendant community. This incident was among many that highlighted the disconnect between the highly heterogeneous African American community whose ancestors were disproportionately represented in these collections and the community of biological anthropologists who were invested in these remains as a source of disciplinary research and teaching materials. We on the Task Force knew that this broader descendant community is one of many, but what we learned from African American participants has resonance with attitudes expressed within the biological anthropology community and, we anticipate, other descendant communities.

Over 3 years, more than 3000 members of the African American community from 14 locations participated in focus groups. From these discussions, we learned that many individuals wanted to have a dialogue with members of the scientific community about the study and stewardship of remains. What emerged were guiding principles, many of which may seem like common sense but have not been common or consistent approaches in the study of human remains:

- Biological anthropologists should follow strict ethical guidelines and protocols in the approach to the stewardship and study of skeletal and other biological remains.
- The regional demographic origins of individuals in a collection should be noted so that the dead can be connected to the living.
- When communicating with descendant communities about the analysis of their ancestors' remains, scientists should follow a culturally sensitive and respectful approach.
- Assumptions should not be made of religious or cultural uniformity in communities.
- Scientists should communicate their findings in language that is clear, accessible, and free of technical jargon.
- Scientists should actively involve descendant communities in the decision‐making process regarding the stewardship and analysis of their ancestors' remains.
- Consent is an ongoing process that requires consistent, clear communication between scientific and descendant communities.
- Scientists must spend enough time in descendant communities to build trust and to become aware of the community's research priorities.
- Research should undergo ethical review by an institutional review board (IRB) or an equivalent ethics committee to ensure that the research is conducted in a responsible and respectful manner.

Data gathered from the biological anthropology community—which took the form of an online survey followed up with individual discussions, listening sessions, and a webinar—indicated that many members of the biological anthropology community support the need to determine provenance (93.7%) and get consent from descendant communities (92%) before conducting research, finding these as essential or of moderate importance. Fewer researchers think that it is at least moderately important that descendant communities should be consulted in planning and interpretation of results (87.7% and 78%, respectively). While the practice of engagement with descendant communities is desired, there are important factors that encumber its practice, including:

- Uncertainty in the provenance of individuals in legacy collections;
- Concerns about how to link provenanced human remains to descendant communities or communities of origin;
- Best practices for conducting research with respect to the wishes of descendant communities; and
- Concerns about having resources (personnel, time, and financial) as well as institutional support to address the Recommendations of the Task Force.

In developing the Recommendations, which should be read in full, we worked to address the desires expressed by the members of the African American community and the biological anthropology community, while also working to find potential solutions that addressed the concerns in implementation noted by biological anthropologists. Like any scientific endeavor, practices and approaches are likely to change with more information and experience. Thus, we anticipate that while we were as thorough as we could be with current information, the AABA will continuously revisit the Recommendations as we enter a new set of practices in the stewardship and study of human remains.

Our surveys reflected great overlap in the perspectives and objectives of the African American community members who participated as well as the scientists who contributed to the study. The establishment and maintenance of trust between descendant communities and researchers is a fundamental theme that emerges from the surveys as well as from the experiences of Task Force members who have engaged in partnered research. Trust comes in many forms, from clear communication about the goals and outcomes of research to the continual provision of agency to the community of origin or descendant community members. The wishes of communities should be recognized and discussed with transparency, and where there may be conflict with the goals of researchers, dialogues should be undertaken with an understanding that there are differences in implicit and explicit power in the relationship between descendants and those who manage collections, excavate sites, or regulate access to remains.

In addition to desiring activities that build trust between descendent communities and scientists as well as educate both communities, both the descendent community and the biological anthropologists appear receptive to mutual engagement to enhance restorative justice and reciprocal education in all components of the research endeavor. This marks a significant shift in the legacy of biological anthropology, one that comes with opportunity as well as, at least for some scientists, uncertainty and concern. Uncertainty can be overcome, though, through community and communication.

To this end, we strongly encourage the AABA Executive Committee to commit to helping members of the AABA and sibling associations address the overlapping priorities of descendant and scientific communities. To avoid a patchwork of implementation, AABA must devote adequate staffing, training, and time for meaningful and consistent collaboration. Only with this equitable approach can we achieve the goals of ethical management and, where desired through mutual agreement, illuminated study of the human remains in legacy collections. The Task Force has developed three potential mechanisms that could lead to minimizing inconsistent approaches by scientists and that are consistent with the expressed desires of the African American communities studied. We are heartened that the first of these, the standing committee, will be emplaced to start implementation of the Recommendations. But the scientific community should strive for the higher‐level mechanisms, which means coordination and cooperation among the many professional associations that represent individuals who work with legacy collections. Again, collective effort and communication can surmount many concerns shared among members of the AABA, as well as give some assurances of consistency to descendant communities.

We know there is more to be addressed and accomplished as recommendations and guidelines are a starting point, and implementation of all or some of these will take time and dedication. There are concerns, such as the few we highlighted as potential encumbrances above, that will take experience, compromise, and diligent conversations to address. Likewise, topics including the sharing and use of data amassed in the study of legacy collections, as well as other sources across the subdisciplines of biological anthropology, await future deliberation. From our study and work, we think that the scientific community is ready to engage in this effort and these dialogues.

Ultimately, we want equitable and fair interactions between descendant communities and research scientists in the future. We are optimistic that continued conversations between biological anthropologists and the broader African American community, among many descendant communities, will create opportunities to beneficially change the way scientists engage with their research, improve the quality of such research, and increase the quantity and significance of biological anthropology studies in both communities. Such efforts do not exculpate the ways in which legacy collections were assembled, but they provide ways forward toward what we hope to be a more ethical and engaged future. Who speaks for the dead will be dependent on how we approach this future and the choices communities make together.

We are grateful to the many individuals who have participated in the work of the Task Force. Thousands of members of the African American community volunteered their time, and their thoughts were instrumental in shaping our efforts. Likewise, candid conversations and insights of members of the scientific community have been invaluable. We thank the members of the Task Force whose sagacity, stamina, and debates strengthened the collective work. Finally, we are grateful to three administrations of the AABA—led by Steve Leigh, then Leslea Hlusko, and finally Anne Stone—for their support and patience, as well as their feedback.

## Author Contributions

**Fatimah L. C. Jackson:** conceptualization, writing – original draft, writing – review and editing. **Benjamin M. Auerbach:** conceptualization, writing – original draft, writing – review and editing.

## Data Availability

Data sharing not applicable to this article as no datasets were generated or analyzed during the current study.
